# A Novel Cellular Pathway of Antigen Presentation and CD4 T Cell Activation *in vivo*

**DOI:** 10.3389/fimmu.2018.02684

**Published:** 2018-11-22

**Authors:** Hannah E. Scales, Gavin R. Meehan, Alan J. Hayes, Robert A. Benson, Emma Watson, Anne Walters, Michio Tomura, Eugene Maraskovsky, Paul Garside, Adriana Baz Morelli, James M. Brewer

**Affiliations:** ^1^Institute of Infection, Immunity and Inflammation, College of Medical, Veterinary and Life Sciences, University of Glasgow, Glasgow, United Kingdom; ^2^CSL Limited, Melbourne, VIC, Australia; ^3^Laboratory of Immunology, Faculty of Pharmacy, Osaka Ohtani University, Tondabayashi, Japan

**Keywords:** antigen processing and presentation, T cell activation, monocytes, subcapsular sinus macrophages, adjuvants, immunologic

## Abstract

Dendritic cell activation of CD4 T cells in the lymph node draining a site of infection or vaccination is widely considered the central event in initiating adaptive immunity. The accepted dogma is that this occurs by stimulating local activation and antigen acquisition by dendritic cells, with subsequent lymph node migration, however the generalizability of this mechanism is unclear. Here we show that in some circumstances antigen can bypass the injection site inflammatory response, draining freely and rapidly to the lymph nodes where it interacts with subcapsular sinus (SCS) macrophages resulting in their death. Debris from these dying SCS macrophages is internalized by monocytes recruited from the circulation. This coordinated response leads to antigen presentation by monocytes and interactions with naïve CD4 T cells that can drive the initiation of T cell and B cell responses. These studies demonstrate an entirely novel pathway leading to initiation of adaptive immune responses *in vivo*.

## Introduction

Here we present a novel cellular pathway of antigen presentation and CD4 T cell activation which challenges the current dogma. T cell responses to peripheral antigen challenge, either against an invading pathogen or vaccination, are initiated in the draining lymph node. A canonical model has been proposed, whereby dendritic cells (DCs) acquire antigen in the tissue and subsequently migrate to the lymph node to interact with and activate T cells (1–3). These processes appear to be influenced by the local inflammatory response in the tissue infection/injection site in response to pathogen and/or damage associated molecular patterns (PAMPS or DAMPS, respectively) (4–6). The combined ability of DCs to sense and respond to challenges in tissues and activate naïve T cells in the lymph node means they have been proposed to have a central role as a key “bridge” between the innate and adaptive immune responses as well as the tissue and the lymphoid system (7–11). However, whether migratory DCs play a role in the initiation of CD4 mediated adaptive immune responses in response to all stimuli remains unclear. For example, their role the mechanism of action of the clinically approved adjuvants such Alum is questioned by the observation that the adaptive immune response is not affected by removal of the injection site 2 h after injection, before significant cell migration can occur (12). Furthermore, small particles typically those less than 100 nm such as Virus Like Particles (e.g., Hepatitis or Papilloma virus vaccines) or soluble antigens may drain freely to the lymph nodes via the lymphatic vessels independently of migratory cells (13–15). These studies suggest that in some cases, CD4 T cell responses may be initiated partially or completely independently of migratory DC populations.

To test this further we employed ISCOMATRIX™ adjuvant (ISCOMATRIX), a novel nanoparticulate adjuvant (40–50 nm diameter) composed of cholesterol, phospholipids and saponin with both immunostimulatory and antigen delivery activity. Immunization with ISCOMATRIX has been shown to induce mixed Th1 and Th2 antibody responses and CD8 T cell responses (16). MHCII has also been shown to be essential for ISCOMATRIX driven CD8 T cells responses (17) therefore CD4 T cells must also be critical for ISCOMATRIX function. However, the mechanism whereby ISCOMATRIX affects antigen presentation on MHCII and activates CD4 T cell responses has not been established. Here, we reveal an alternative pathway for the induction of adaptive immune responses by demonstrating that ISCOMATRIX induced inflammation at the site of inoculation is not required for the induction of the adaptive immune response but rather ISCOMATRIX induces inflammation and cell death directly within the draining lymph node. We subsequently show that, rather than migratory DCs, the important cell type presenting antigen on MHCII to CD4 T cells are monocytes that have been recruited directly to the lymph node following immunization.

## Results

### Co-administered soluble antigen and adjuvant are not retained at the injection site, draining rapidly to the lymph node

**Figure 1:**
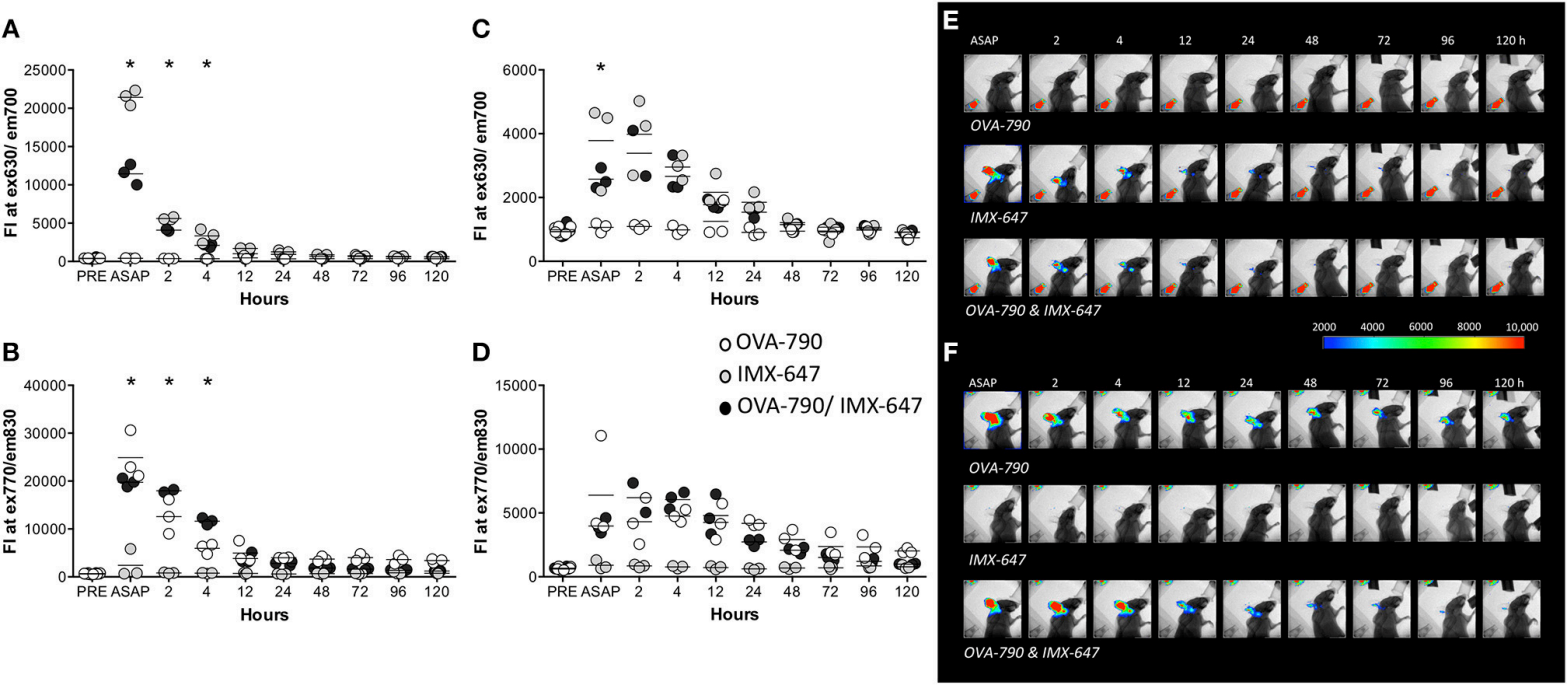
ISCOMATRIX does not alter the passage of soluble antigen from the injection site to the lymph node. Graphs show fluorescence intensity of **(A)** ISCOMATRIX-647 detected at 630 ± 17.5 nm and **(B)** OVA-790 detected at 830 ± 17.5 nm in the ear pinnae and **(C)** ISCOMATRIX-647 and **(D)** OVA-790 in the draining cervical lymph node over time. Representative X-ray images with pseudocoloured fluorescence images overlaid showing **(E)** ISCOMATRIX-647 fluorescence (630 ± 17.5 nm) and **(F)** OVA-790 fluorescence (830 ± 17.5 nm) over time following injection in the ear pinnae with OVA-790 alone, ISCOMATRIX-647 alone or OVA-790/ISCOMATRIX-647. Three animals were imaged at each time point. IMX = ISCOMATRIX™ adjuvant, ^*^P < 0.05.

It has been suggested that the prolonged presence of antigen is important in driving an adaptive immune response, with the formation of an antigen depot at the injection site often considered to be a key element in the mode of action of adjuvants (18, 19). We therefore utilized whole body imaging of mice following the injection of fluorescently labeled antigen (OVA-790) and/ or ISCOMATRIX (ISCOMATRIX-647) into the ear pinnae of C57BL/6 mice to track the location of a soluble antigen and adjuvant. Image analysis demonstrated that the presence of antigen in the ear is not prolonged by ISCOMATRIX with the fluorescence intensity of both antigen and adjuvant returning to near background levels within 24 h (Figures 1A,B,E,F). OVA-790 and ISCOMATRIX-647 were rapidly displaced to the superficial cervical lymph node draining the ear within 15 min of injection. The fluorescence signal from both OVA-790 and ISCOMATRIX-647 remained detectable within the lymph node for 48 h, the presence of ISCOMATRIX however does not alter the magnitude or duration of the OVA-790 signal (Figures 1C,D,E,F). These data suggest that ISCOMATRIX does not form an antigen depot at the injection site or in the draining lymph node.

### Neutrophils and monocytes are recruited to the antigen/adjuvant injection site and draining lymph node

**Figure 2:**
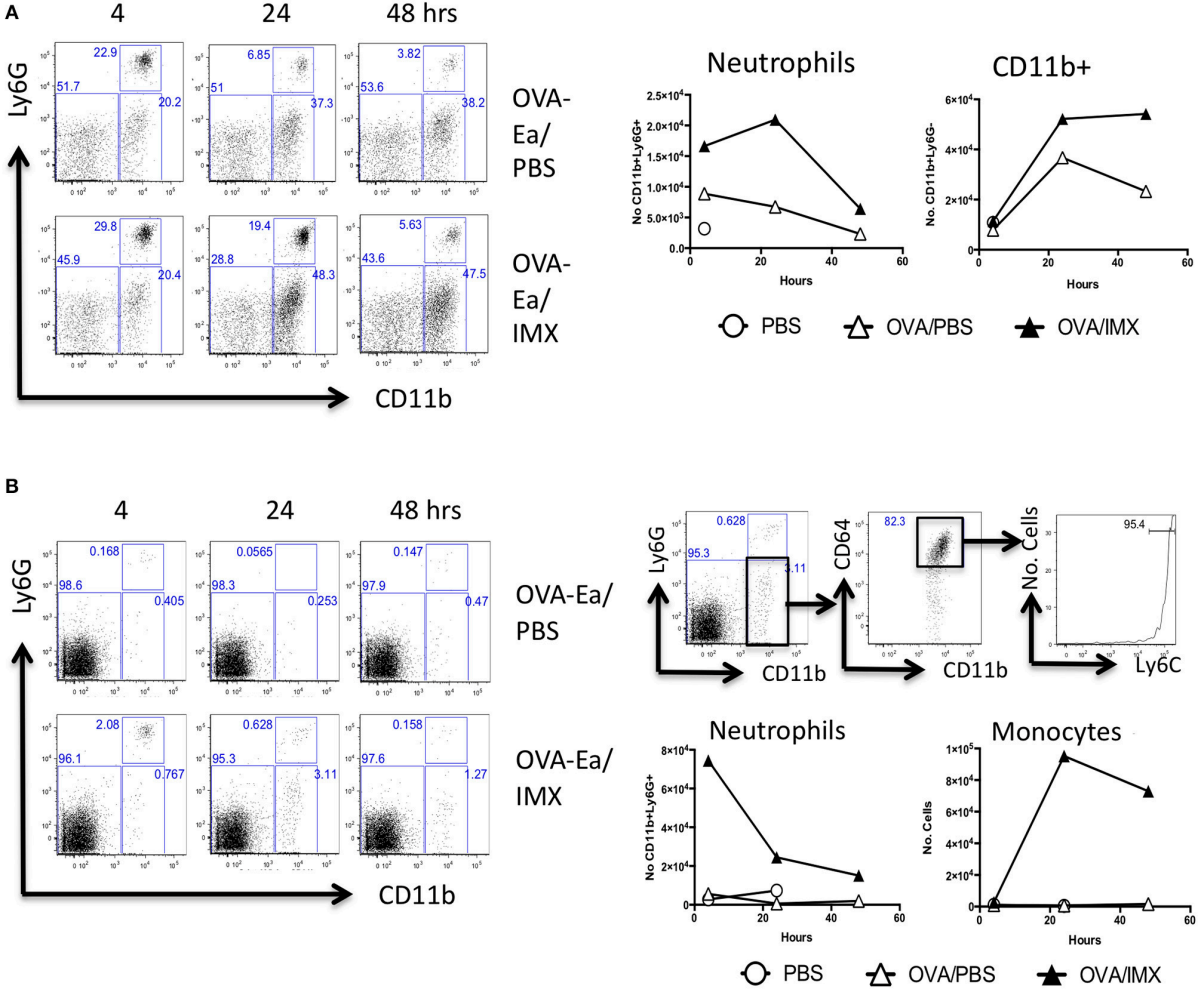
Neutrophils and CD11b+ cells are recruited to both the injection site and to the draining lymph node. **(A)** Representative flow cytometry plots showing the recruitment of neutrophils and CD11b+ cells in the injection site at 4, 24, and 48 h following immunization with OVA/PBS (top) or OVA/ISCOMATRIX (bottom). Absolute numbers are shown in the graphs on the right. **(B)** Representative flow cytometry plots showing the recruitment of neutrophils and CD11b+ cells in the draining cervical lymph node at 4, 24, and 48 h following immunization with OVA/PBS (top left) or OVA/ISCOMATRIX (bottom left). Further representative plots show the identification of the majority of the CD11b cells in the OVA/ISCOMATRIX treated lymph node at 24 h were monocytes (CD64+Ly6C high) (top right). Absolute numbers of Neutrophils and monocytes recruited to the draining lymph node in response to OVA/PBS and OVA/ISCOMATRIX are shown (bottom left). Data shown is from 3 mice per group pooled and is representative of 5 independent experiments. IMX = ISCOMATRIX™ adjuvant.

Localized inflammation at the injection site may also enhance the development of an adaptive immune response in the draining lymph node following immunization (17, 20). Compared with injection with OVA alone, ear pinnae injected with OVA-ISCOMATRIX demonstrated a significant early neutrophil influx, starting at 4 h and peaking at 24. This was followed by an influx of CD11b+ cells, presumably monocytes at 24 and 48 h (Figure 2A). Analysis of the lymph node draining the ear showed that ISCOMATRIX stimulated a similar but shorter-lived infiltration of neutrophils at 4 h followed by an increase in CD11b+ cells. Further analysis revealed the majority of CD11b+ infiltrating cells were CD64+Ly6Chi monocytes, their numbers peaking at 24 h and beginning to decline by 48 h (Figure 2B).

### Antigen/adjuvant injection site inflammation does not contribute to cell migration or activation of immune responses in the draining lymph node

**Figure 3:**
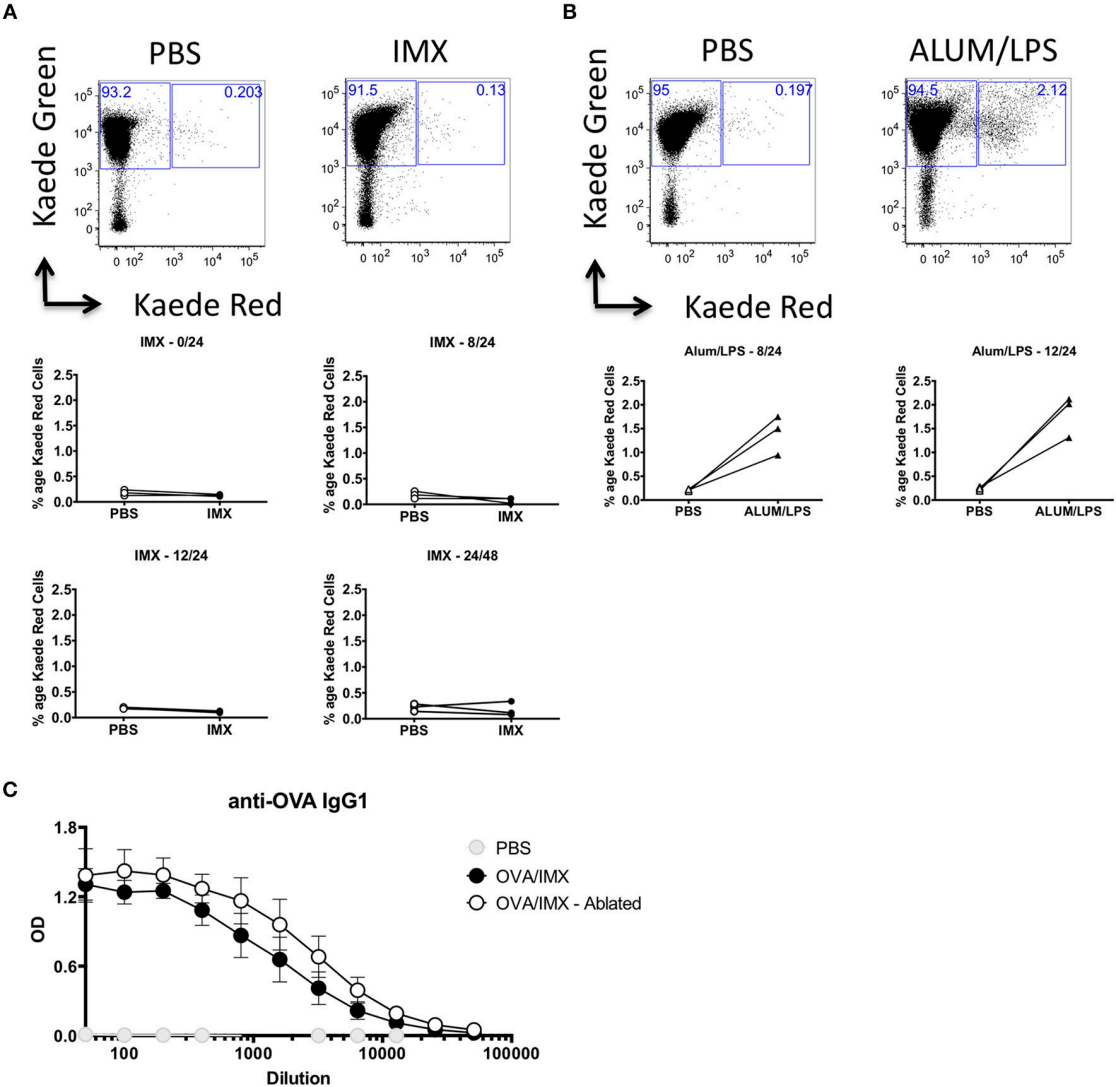
The injection site is not required for ISCOMATRIX adjuvant activity. **(A)** Representative flow cytometry plots showing Kaede red vs. Kaede green in the popliteal lymph node (top panels) draining the PBS injected (left) and the ISCOMATRIX (right) injected footpad, below plots showing the percentage of the leukocytes in the draining lymph nodes at 24 h following photoswitching immediately prior (middle left), 8 h post (middle right) 12 h post (bottom left) immunization or at 48 h from mice photoswitched at 24 h (bottom right). **(B)** Representative flow cytometry plots showing Kaede red vs. Kaede green in the popliteal lymph node (top panels) draining the PBS injected (left) and the ALUM/LPS (right) injected footpad, below plots showing the percentage of the leukocytes in the draining lymph nodes at 24 h following photoswitching 8 h post (bottom left) and 12 h post (bottom right) immunization. **(C)** Serum anti-OVA IgG1 from mice at 14 days post immunization with PBS, OVA/ISCOMATRIX with the injection site intact or OVA/ISCOMATRIX with the injection site removed shortly following immunization. Groups contained 3 animals and data is representative of two independent experiments. IMX = ISCOMATRIX™ adjuvant.

Injection site inflammation is thought to drive skin resident DC migration and recruitment of inflammatory cells that may subsequently migrate to the lymph node (20). In order to definitively identify cells migrating from the injection site to the draining lymph node, we utilized the Kaede transgenic mouse. These mice ubiquitously express the Kaede fluorescent protein that normally emits in the green spectrum (518 nm), however Kaede can undergo UV induced fission to a form that fluoresces red (582 nm) (21). We immunized Kaede mice in the left footpad, with PBS and ISCOMATRIX on the right. Both feet were photoswitched using a UV laser either prior to immunization or 8, 12, or 24 h post-immunization. The draining lymph node was then analyzed for the presence of injection site derived, Kaede red cells at 24 or 48 h post-immunization. Mice immunized with Alum/LPS were used as a positive control. In ISCOMATRIX treated tissue no increase in the number of red cells was observed compared with the PBS treated control lymph node at any time point tested suggesting that ISCOMATRIX does not stimulate cell migration from the injection site to the lymph node (Figure 3A). We could clearly identify an increase in the percentage of Kaede red cells in the draining lymph node of mice immunized with the positive control, Alum/LPS at both time points assessed (Figure 3B). While these studies definitively confirmed that ISCOMATRIX does not stimulate cell migration from the injection site to the lymph node, the inflammatory environment identified at the injection site could still contribute to or influence the immune response developing within the lymph node, for example via soluble mediators (22, 23). To analyse this, we immunized mice with OVA/ISCOMATRIX and then performed injection site removal. Ablation of the injection site within 15 min of the administration of OVA/ISCOMATRIX failed to significantly alter antigen-specific IgG1 levels at 14 days (Figure 3C) demonstrating that events at injection site are not required for ISCOMATRIX adjuvant activity and are therefore not a prerequisite to drive adaptive immune responses to antigen.

### Antigen/adjuvant localizes to the subcapsular sinus (SCS) and causes loss of CD169+ macrophages

**Figure 4:**
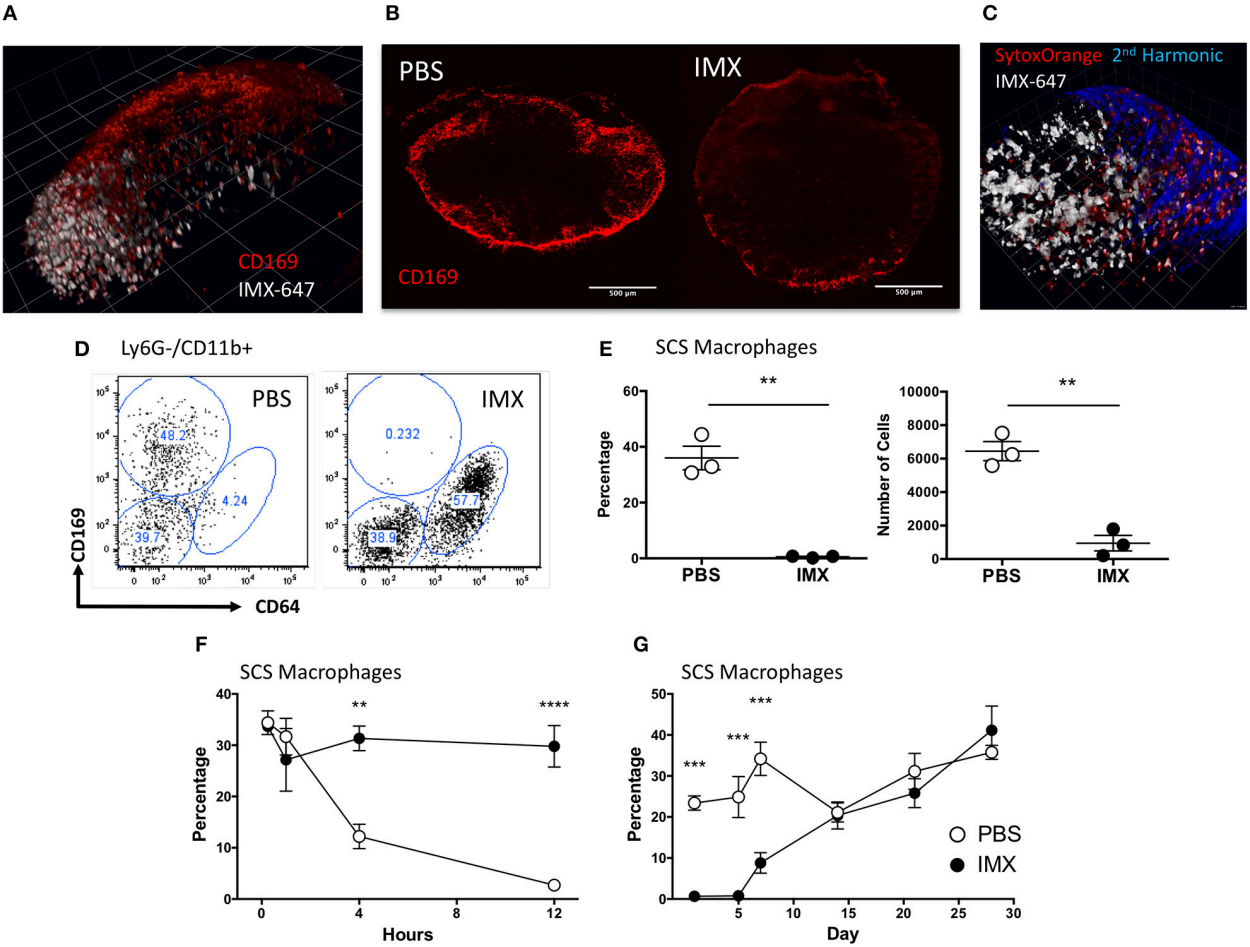
ISCOMATRIX localizes to the subcapsular sinus macrophages and these cells are lost from the lymph node following ISCOMATRIX treatment. **(A)** Mulitphoton image showing a popliteal lymph node labeled *in vivo* with a PE conjugated anti CD169 antibody and subsequently treated with 1 IU ISCOMATRIX-647 in the footpad. Imaging was performed 1 h after treatment with the ISCOMATRIX-647. **(B)** CD169 Immunohistochemical staining of cervical lymph nodes 24 h following immunization in the ear pinnae with either PBS or ISCOMATRIX. **(C)** Mulitphoton image showing the popliteal lymph node approximately 2 h following the co-administration of the cell impermeable DNA dye Sytox orange and ISCOMATRIX-647 in the footpad. **(D)** Representative plots showing CD169 and CD64 expression on Ly6G- CD11b+ cells in the draining lymph node from mice treated with PBS or ISCOMATRIX at 24 h. **(E)** Flow cytometry data showing the percentage and number of CD11b+ cells that are CD169+ SCS macrophages in the cervical lymph node at 24 h following immunization with PBS or ISCOMATRIX in the ear pinnae. **(F)** The percentage of CD11b+ cells that are CD169+ SCS macrophages in the cervical lymph node over the first 12 h following immunization with PBS or ISCOMATRIX in the ear pinnae. **(G)** The recovery of the of CD11b+ cells that are CD169 + SCS macrophages in the cervical lymph node over 28 days following immunization with PBS or ISCOMATRIX in the ear pinnae. Groups contained 3 animals. IMX, ISCOMATRIX™ adjuvant, ^**^P < 0.01, ^***^P < 0.001 ^***^P < 0.0001.

The studies above demonstrate that neither tissue resident cells nor the inflammatory reaction induced by adjuvant at the injection site are required to develop an adaptive immune response to co-administered antigen. Therefore, following the rapid displacement of antigen and ISCOMATRIX to the draining lymph node, the events occurring within the draining lymph node appear sufficient to drive an adaptive response. To localize ISCOMATRIX within the draining lymph node we administered a PE labeled anti-CD169 antibody to mice via the footpad to label subcapsular sinus (SCS) macrophages in the draining popliteal lymph node. Approximately 1 h after immunization with ISCOMATRIX-647-OVA, intravital imaging revealed adjuvant localization to the sinus region of the lymph node and colocalization with CD169+ SCS macrophages (Figure 4A). Subsequently, analysis of lymph node tissue sections prepared 24 h after ISCOMATRIX immunization demonstrated a reduction in the SCS macrophage population compared with PBS treated controls (Figure 4B). Intravital imaging at approximately 2 h following administration of the cell impermeable DNA dye, Sytox Orange, showed that cells labeled with ISCOMATRIX-647 were also positive for the Sytox Orange suggesting that these cells were dying (Figure 4C). Flow cytometry confirmed an almost complete absence of CD11b+ CD169+ cells 24 h post ISCOMATRIX immunization (Figures 4D,E). Kinetic analysis demonstrated that loss of SCS macrophages (CD11b+ CD169+) began as soon as 4 h post-immunization with ISCOMATRIX, with an almost total absence by 12 h (Figure 4F). A significant reduction in the percentage of these cells was maintained until 14 days post-immunization when they had recovered to levels similar to that observed in the contralateral ear draining lymph node (Figure 4G).

### SCS macrophages killed by adjuvant action are taken up by monocytes recruited to T cell dependent areas

**Figure 5:**
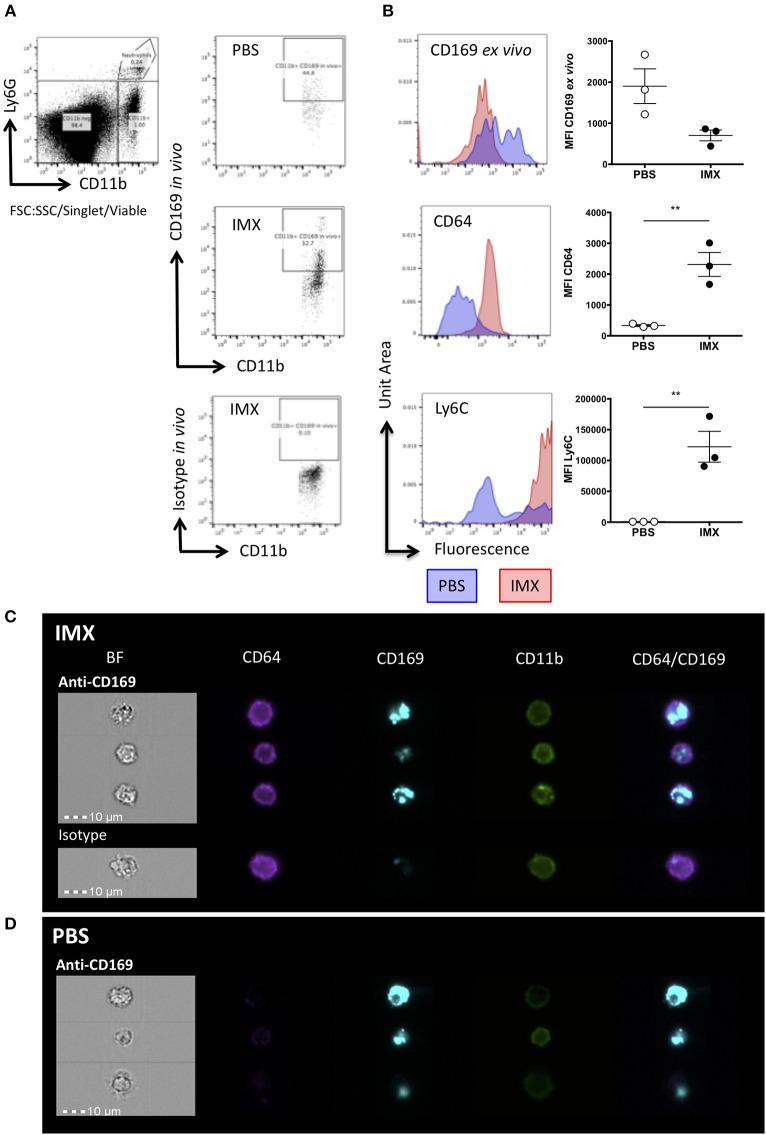
SCS macrophages are taken up by monocytes following immunization with ISCOMATRIX. **(A)** Representative flow cytometry plots showing *in vivo* antibody staining with eFluor660 conjugated anti-CD169 prior to treatment with PBS (top) and ISCOMATRIX (middle) or the isotype control antibody (bottom) treatment in the ear pinnae. **(B)** Representative histograms and MFI (points = individual animals, lines show mean±SEM) of *ex vivo* PE conjugated anti-CD169 (top); anti-CD64 (middle), and anti-Ly6C (bottom) on cells positive for the *in vivo* CD169 label. Groups contained 3 animals and data is representative of two independent experiments ^**^P < 0.01. **(C)** Representative imaging flow cytometry images showing monocytes at 24 h post-immunization with ISCOMATRIX from the lymph nodes of mice following *in vivo* eFluor660 conjugated anti-CD169 (top) or the isotype control (bottom) labeling. **(D)** Representative imaging flow cytometry images showing macrophages (CD11b+) at 24 h post-immunization with PBS from the lymph nodes of mice following *in vivo* eFluor660 conjugated anti-CD169 labeling. The anti-CD169 antibody treated group contained 3 animals, while the isotype control treated group contained 2 animals. IMX = ISCOMATRIX™ adjuvant.

To identify where SCS macrophages and/or their debris traffic to following immunization, we labeled them *in vivo* with a fluorescently labeled anti-CD169 (eFluor660) antibody prior to immunization. Twenty-four hours later, the draining lymph nodes were harvested and stained *ex vivo* with antibodies to CD11b, CD64, Ly6C, and CD169 (conjugated to PE) followed by flow cytometric analysis. At 24 h post-immunization with ISCOMATRIX, the CD169 *in vivo* label (Figure 5A) was found in cells that were *ex vivo* CD169- but CD64+ and Ly6C hi (Figure 5B). By contrast, the CD169 *in vivo* positive cells (Figure 5A) from PBS treated lymph node were *ex vivo* CD169 positive, CD64-and Ly6C neg-low (Figure 5B). Furthermore, CD11b-CD11c+ DCs at 4 h post-immunization with ISCOMATRIX acquire the CD169 *in vivo* label at levels similar to that observed in the PBS treated mice (Supplementary Figure 1A), by 24 h the ISCOMATRIX treated mice have lost the CD169 *in vivo* label from their CD11b-CD11c+ DCs (Supplementary Figure 1B). The degree of CD169 *in vivo* fluorescence labeling in these DCs is substantially lower than that observed in the CD11b+CD64+ monocytes (Supplementary Figure 1C).

ImageStream analysis of *in vivo* anti-CD169 eFluor660 labeled lymph nodes at 24 h post-immunization with ISCOMATRIX shows the e660 label was present in internal compartments of CD64+CD11b+ cells (Figure 5C), while in mice treated with the PBS control the e660 label was detected predominantly in CD64-CD11b+ cells suggesting that the SCS macrophages had taken up the antibody label directly (Figure 5D).

This data suggests that following treatment with ISCOMATRIX, CD169+ SCS macrophages die and are taken up by monocytes. We hypothesized that dying SCS macrophages could carry antigen and may furthermore be a source of DAMPS within the lymph node providing an inflammatory signal to facilitate T cell activation.

### Adjuvant can promote, monocyte acquisition, processing and presentation of antigen in the context of MHCII

**Figure 6:**
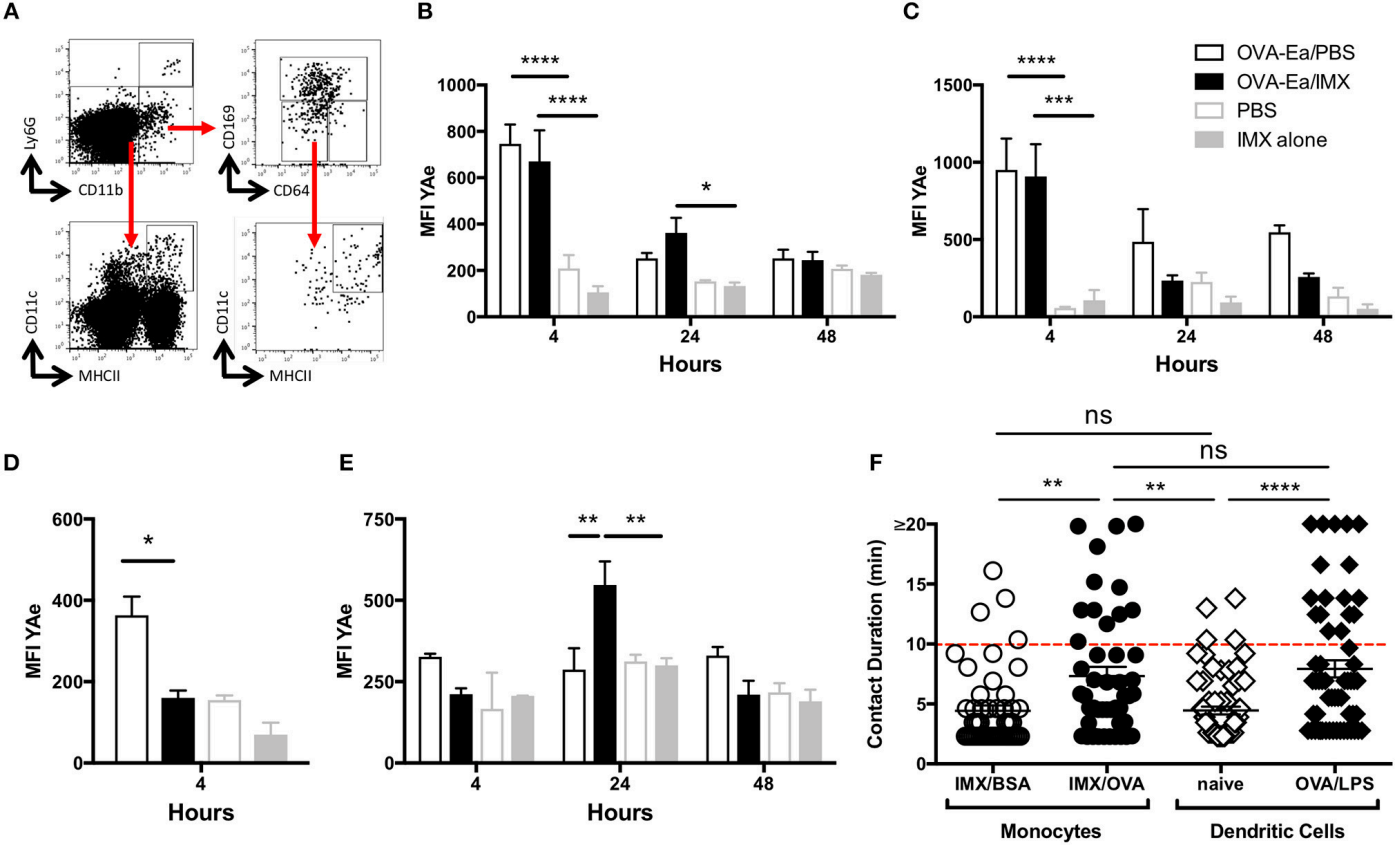
Recruited monocytes present antigen and may be observed interacting with T cells in the lymph node at 24 h post-immunization. **(A)** Representative plots showing flow cytometry gating strategy for the identification of CD11b+CD64+ monocytes, for CD11b+CD169+ SCS macrophages and CD11b- or CD11b+ CD11c+MHCII+ dendritic cells. Graphs showing mean (±SEM) YAe MFI on CD11b- **(B)** and CD11b+ **(C)** dendritic cells at 4, 24, and 48 h; mean (±SEM) YAe MFI SCS macrophages **(D)** at 4 h; mean (±SEM) YAe MFI on monocytes **(E)** at 4, 24, and 48 h. Groups contained 3 animals and data is representative of three independent experiments. **(F)** LysM-EGFP or CD11cYFP mice were immunized in the footpad (OVA or BSA/ISCOMATRIX or OVA/LPS respectively) ~20 h later the draining popliteal lymph node was surgically exposed. OTII DSRed T cells and monocytes (GFP+) or DCs (YFP+) were imaged by live real time multiphoton microscopy. Interaction between T cells and monocytes (and T cells and DCs was identified by colocalization of the DSRed signal with the GFP or YFP signals and tracked for up to 20 min, 48 such interactions were tracked in the OVA/ISCOMATRIX treated mice. IMX = ISCOMATRIX™ adjuvant, ^*^P < 0.05, ^**^P < 0.01, ^***^P < 0.001 ^****^P < 0.0001.

Classically, antigen presentation to T cells in the lymph node was thought to be performed by DCs (1, 9, 24). However, we previously observed (Figure 2) that OVA/ISCOMATRIX stimulates significant recruitment of monocytes to the lymph node, which were clearly not injection site derived (Figure 3). To investigate the role of monocytes in the induction of T cell responses triggered by ISCOMATRIX we used the E-alpha: YAe model, where presentation of the peptide on MHCII may be detected by flow cytometry using the YAe antibody (Supplementary Figure 2) (1). Mice were immunized with the E-alpha peptide conjugated to OVA (OVA-Eα) with or without ISCOMATRIX and mice immunized with PBS or ISCOMATRIX alone were used as controls. The populations of monocytes, DCs and SCS macrophages were identified (Figure 6A) and the degree of antigen presentation on these populations assessed by YAe staining. At 4 h post-immunization, antigen presentation was primarily detected on CD11b-CD11c+ and CD11b+CD11c+ DCs in both the presence and the absence of ISCOMATRIX (Figures 6B,C). Antigen presentation was also observed on CD169+ SCS macrophages at 4 h, however this was only detectable in the absence of ISCOMATRIX (Figure 6D). Antigen presentation waned rapidly in the absence of ISCOMATRIX, and was no longer detectable 24 h after immunization (Figures 6B,C). By contrast, ISCOMATRIX and OVA-Eα immunization resulted in antigen presentation by CD11b+CD64+ monocytes that actually increased at 24 h (Figure 6E). To identify which cell population was functionally presenting antigen to T cells, we performed *in vivo* multiphoton imaging of OTII DSRed T cells in the popliteal lymph node of LysM-EGFP mice 24 h following immunization with OVA and ISCOMATRIX. While GFP is strongly expressed in neutrophils in these mice, it is also expressed in monocytes (Supplementary Figures 3A,B), which are more numerous in the draining lymph node 24 h post ISCOMATRIX immunization (Figure 2) in addition monocytes express higher levels of MHCII relative to neutrophils (Supplementary Figure 3C). These images show that following immunization with OVA/ISCOMATRIX, antigen-specific T cells interact with monocytes in the draining lymph node (Supplementary Video 1). Previous studies by both our laboratory and by others have demonstrated that the duration of T cell interactions with APCs is functionally important in driving T cell responses, with longer duration interactions driving stronger responses (25–29). Here the comparison of T cell monocyte interaction duration between ISCOMATRIX immunized and unimmunized animals is not possible due to the low numbers of monocytes present within lymph nodes. However, analysis of T cell—monocyte interaction duration in OVA/ISCOMATRIX immunized lymph nodes showed a similar distribution of interaction lengths as is observed between T cells and CD11c+ DCs following immunization with OVA and LPS at ~20 h (Figure 6F, Supplementary Video 1). While the T cell—monocyte interaction duration following immunization with ISCOMATRIX and bovine serum albumin (a non-cognate antigen for OTII T cells) and the interaction duration between T cells and CD11c+ DC in naïve mice are also similar (Figure 6F). Collectively, these data suggest the interactions between T cells and monocytes that formed after immunization with OVA/ISCOMATRIX were both antigen specific and functional.

### A novel cellular pathway of antigen presentation stimulates robust Tfh and germinal center B cell responses

**Figure 7:**
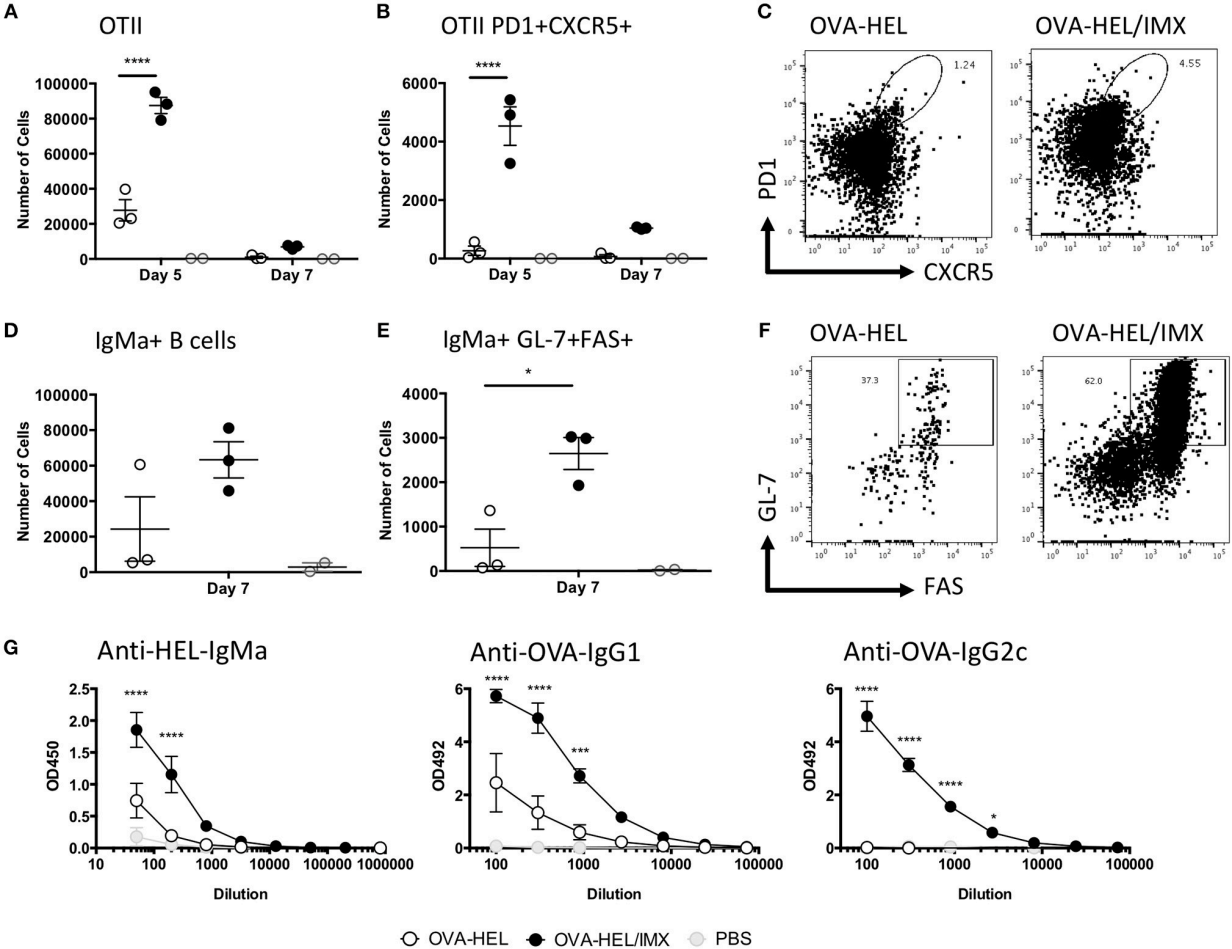
ISCOMATRIX stimulates Tfh and germinal center B cell responses. **(A)** Total number of OVA specific (OTII) CD4+ T cells in the lymph node, and **(B)** the number are CXCR5+PD1+ at 5 and 7 days. **(C)** Representative flow cytometry plots showing CXCR5 and PD1 staining on OTII CD4+ T cells from OVA-HEL and OVA-HEL/ISCOMATRIX treated mice at 5 days. **(D)** Total number of HEL specific (MD4) B cells and **(E)** the number that are GL-7+/FAS+ at 7 days. **(F)** Representative flow cytometry plots showing GL-7 and FAS staining on MD4 B cells from OVA-HEL and OVA-HEL/ISCOMATRIX treated mice at 7 days. **(G)** Graphs showing the mean ± SEM serum anti-HEL IgMa, anti-OVA IgG1, and anti-OVA IgG2c titers at 14 days post-immunization with OVA-HEL, OVA-HEL/ISCOMATRIX or PBS. Immunized groups contained 3 animals and are representative of 2 independent experiments. IMX = ISCOMATRIX™ adjuvant, ^*^P < 0.05, ^***^P < 0.001, ^****^P < 0.0001.

We have shown that the recruited monocytes are able to form interactions with CD4+ T cells and although it has previously been shown that there is a requirement for CD4+ T cells in the development of both ISCOMATRIX driven CD8+ T cell and antibody responses *in vivo* (17) it is not known how the CD4+ T cell response develops following antigen presentation by monocytes. We therefore utilized a transgenic TcR and BcR cell adoptive transfer model to determine the effect of ISCOMATRIX on this aspect of the immune response (30). Following adoptive transfer of OVA-specific CD4+ OTII T cells and HEL-specific MD4 B cells, recipient mice were immunized with HEL-OVA in the presence or absence of ISCOMATRIX. Five days after immunization, OVA-HEL co-administered with ISCOMATRIX induced a significant expansion of OVA-specific OTII T cells compared with antigen administered alone (Figure 7A). A significant increase in numbers of antigen specific Tfh (PD1+ CXCR5+) was also observed at day 5 (Figures 7B,C); while by day 7 an increased number of HEL specific germinal center B cells was observed in mice receiving ISCOMATRIX compared with antigen alone (Figures 7D,E,F). By day 14 this had translated into ISCOMATRIX driven increases in serum titres of HEL specific IgMa and of OVA specific IgG1 and IgG2c (Figure 7G).

## Discussion

The current studies demonstrate unequivocally that the adjuvant co-administered with an antigen can obviate the requirement for the skin to lymph node migration of tissue resident or recruited DCs, or other innate cells to elicit an antigen specific CD4 T cell responses. We have in fact discovered an entirely novel pathway whereby antigen bearing SCS macrophages are killed by the associated adjuvant, internalized by monocytes recruited from the circulation and it is these cells that appear to respond to ISCOMATRIX adjuvant by increasing antigen presentation to naïve CD4 T cells resulting in a robust antibody response. This finding has important implications for vaccine design and delivery.

The activation of, and acquisition of antigen by DCs in peripheral tissues followed by their migration to lymph nodes is considered a key step in the induction of an adaptive immune response (1, 3). The ability of adjuvants to initiate or enhance this process is thought to underlie their immunostimulatory function (18). Using the photoconvertable (Kaede) mouse model (21), we demonstrated that injection of antigen and ISCOMATRIX fails to induce skin to lymph node migration of any cells, including DCs. Furthermore, tissue ablation demonstrated no role for cells and mediators resident or induced at the injection site in mediating the adjuvant activity of ISCOMATRIX. By contrast, we have recently shown that the very rapid (< 2 h) inflammatory response induced at the injection site by Alum adjuvant does play a role in adjuvant activity, through generation of neutrophil NETS which subsequently affect T and B cell responses in the draining lymph node (31). In the case of ISCOMATRIX, the lack of a requirement for tissue inflammatory responses was consistent with the rapid translocation of antigen and adjuvant to the draining lymph node, which was almost complete 15 min after injection. This rapid displacement of ISCOMATRIX to the draining lymph node can be explained by the physical nature of this adjuvant. While particles >200 nm generally require clearance by migratory phagocytes, nanoparticles in the range 10–200 nm can directly enter lymphatic vessels by diffusion through flap valves between lymphatic endothelial cells (32). ISCOMATRIX particles are ~40–50 nm in diameter (33), suggesting their rapid exit from the injection site may occur via this pathway. After entry into lymphatic vessels, fluid and small particles subsequently enter the lymph node in the SCS (13, 15). Soluble material, such as proteins < 70 kDa can directly access the T cell areas of the lymph node through entry into the fibroblastic reticular cell (FRC) conduit system (34). By contrast, larger particles, including bacteria and viruses are retained in the SCS and are sampled by CD169+ macrophages (35, 36). Intravital imaging demonstrated that ISCOMATRIX accumulates in SCS macrophages and resulted in cell killing, producing almost complete depletion of this cell population within 12 h of ISCOMATRIX administration. This *in vivo* observation is consistent with previous *in vitro* data showing ISCOMATRIX internalization directly results in inflammasome activation, and subsequent death of peritoneal macrophages (37). Recently, inflammatory stimuli such as TLR9 agonists and vaccinia virus have been shown to mediate loss of the SCS macrophage population in the lymph node (38). Furthermore, it has been shown that vaccinia virus induces inflammasome activation and death of SCS macrophages by pyroptosis. This results in release of extracellular ASC specks which drive lymph node inflammation (39), while other work using an attenuated influenza vaccine has shown a role for TLR7 signaling in the death of SCS macrophages and subsequent IL-1α release (40). The previously shown partial reduction of ISCOMATRIX adjuvant activity in ASC and caspase- 1/11 deficient mice, and the ISCOMATRIX driven *in vitro* death of macrophages in combination with our data suggests that SCS macrophages may be a key point where innate immune activation plays a role in mediating ISCOMATRIX adjuvant activity (37).

At this point our own studies and those described failed to explain how pyroptosis of SCS macrophages could lead to antigen presentation to naïve T cells. Surprisingly, labeling of SCS macrophages conclusively demonstrated that cell debris was not taken up by DCs, but by CD64+/Ly6C+ monocytes that were recruited to the lymph node in response to ISCOMATRIX treatment. We furthermore confirmed that these recruited monocytes not only presented antigen but also formed cognate interactions with antigen specific CD4+ T cells. While these studies do not disprove a role for lymph node resident DC in antigen presentation, the increase in antigen presentation by monocytes, and not DC induced by adjuvant, suggests these cells are responsible, at least in part for ISCOMATRIX adjuvant activity.

The canonical model of T cell activation via the migration of activated DCs from the site of injection to the draining lymph node has relevance to some immunostimulatory adjuvants. However, given the broad range of agents with adjuvant activity, such a model was unlikely to explain how all adjuvants work. Furthermore, a clear outcome from this model was that adjuvant activity required initiation of injection site inflammation to drive recruitment and/or migration of DCs from the tissue. This is clearly a challenge to the production of vaccines that minimize injection site reactions and associated pain. Therefore, an ideal adjuvant would target immune interactions in the draining lymph node, generating good antigen specific T and B cell responses, while avoiding injection site reactions. While we identified that ISCOMATRIX does induce local inflammation, this was dispensable for adjuvant action. In dissecting the underlying mechanism that allows ISCOMATRIX to drive adjuvant activity in the lymph node, we have identified an entirely novel pathway that contrasts with the existing dogma explaining the initiation and enhancement of the immune response.

## Materials and methods

### Mice

Seven to Ten week-old male C57BL/6J mice were purchased from Charles River Laboratories (Bicester UK). OT-II TCR Tg mice (41) and MD4 BcR Tg mice (42) on C57BL/6 backgrounds were used as a source of OVA specific T cells and HEL specific B cells respectively. Kaede mice were obtained from Michio Tomura (Yokohama); these mice have the Kaede fluorescent protein green knocked in under the control of a synthetic beta-actin promoter. As a result all cells express the photoconvertable kaede protein (21). LysM-EGFP mice were a kind gift from Professor Sussan Noursargh. These mice have the gene for EGFP knocked into the Lysozyme (Lys) M locus resulting in mice with fluorescent myelomonocytic cells, with neutrophils being EGFP^hi^ and monocytes EGFP^low^. CD11cYFP mice express YFP under the control of the CD11c promoter resulting in YFP expression in all CD11c+ cells (43). hCD2-DsRed mice (gifted by D Kioussis and A Patel, National Institute for Medical Research, London) were crossed with OVA specific OT-II (41) TCR Tg mice.

Animals were maintained under standard animal house conditions at the University of Glasgow and procedures were performed under a UK Home Office license in accordance with UK Home Office regulations following review by the University of Glasgow Ethics Committee.

### Immunization, adjuvant, and antigens

All mice were either injected subcutaneously (s/c) in the ear pinna with 10 μL or in the footpad 25 μL of antigen/adjuvant solutions. Mice were immunized with either 100 μg of chromatographically purified chicken ovalbumin (OVA; Worthington Biochemical, Lakewood, NJ, USA); 100 μg of bovine serum albumin (BSA; Sigma Aldrich, Dorset, UK); with 50 μg OVA-conjugated to the Eα peptide (ALMAC, Scotland, UK); for the whole-body imaging experiments mice were immunized with 30 μg OVA conjugated to AlexaFluor 790, this was prepared according to the manufacturer's instructions using the AlexaFluor790 NHS ester (ThermoFisher). In other experiments mice were immunized with 50 μg OVA-HEL conjugate prepared using glutaraldehyde to couple the chicken OVA and HEL (Sigma Aldrich, Dorset, UK) as previously published (30). Antigens were given with 1 IU ISCOMATRIX (CSL Limited, Australia), in some experiments or some control animals received ISCOMATRIX alone. In other experiments ISCOMATRIX labeled with the fluorescent dyes AlexaFluor 647 (CSL Limited, Australia) was used. For the identification of dying cells Sytox Orange (ThermoFisher) was administered with ISCOMATRIX. Control mice for the cell migration experiment were injected with 1% Alum suspension (a kind gift from Dr Erik Lindblad, Brenntag Biosector, Denmark) and 8 μg LPS (*E. coli* 0111:B4; Sigma Aldrich, UK).

### Flow cytometry

Ear tissue was cut up into ~2 mm × 2 mm pieces and digested in 2 mg/mL Collagenase IV; 2 mg/mL Hyaluronidase (both Sigma Aldrich) and 100 U/mL DNaseI (ThermoFisher Scientific) at 37°C for 30 min. Following digestion tissue was homogenized using a gentleMACs Dissociator (Miltenyi Biotech, UK). The draining superficial cervical or popliteal lymph nodes were gently mashed against nitex (Cadisch Precision Meshes, UK) using the rubber end of a syringe plunger and digested in 2.68 mg/mL collagenase D (Roche) for 25 min at 37°C. The enzymatic reaction was stopped by adding EDTA to a final concentration of 10 mM. A single cell suspension was then obtained by passing the lymph node or tissue suspensions through a 70 μm cell strainer. Single cell suspensions were then stained with combinations of the following antibodies: anti-CD11c (N418;), anti-CD11b (M1/70), anti-CD45 (30-F11), anti-MHCII (M5/114.15.2), anti-CD169 (SER4); anti-B220 (RA3-6B2) (all eBioscience, Hartfield, UK); or anti-GL-7 (GL7), anti-FAS-PE-Cy7 (Jo2) anti-CD45.1 (A20), anti-Vb5.1/5.2 (MR9-4) (all BD Biosciences,) or anti-CD4 (GK1.5), anti-CD64 (X54-5/7.1), and anti-Ly6-G (IA8) (all BioLegend, London, UK), conjugated to the following fluorochromes as appropriate Alexafluor488, AlexaFluor647, APC, APC-Cy7, APC-eFluor780; Brilliant Violet 421, eFluor450, eFluor660, eVolve605, FITC, PE, PE-Cy5, PE-Cy7, PERCPCy5.5 or PERCP-eFluor710. Biotinylated antibodies against YAe (YAe-eBio; eBiosciences) and IgMa (DS-1; BD Biosciences) were also used where appropriate and detected with either Streptavidin APC or APC-eFluor780 (eBiosciences) as appropriate. Antibody staining was performed in conditioned media from the anti-CD16/CD32 antibody producing hybridoma (2.4G2) (FcBlock). The viability of the cells was confirmed using fixable eFluor450, eFluor506, or eFluor780 viability dyes (eBioscience). Samples were acquired using a LSRII flow cytometer running FACDiva software (both BDBiosciences) and or on a MACSQuant flow cytometer (Miltenyi Biotech, UK). Analysis was performed using FlowJo software (Version 10.0.8; Treestar).

### Enzyme linked immunosorbant assay (ELISA)

HEL specific IgMa or OVA specific IgG_1_ and IgG_2a_/IgG_2c_ titres were determined in serum samples collected 14 and 21 days after immunization as previously described (44).

### Whole body *in vivo* optical imaging

Images were acquired on a Kodak *in vivo* imaging system FX Pro (Carestream, Hertfordshire, UK). Animals were anesthetized using an isoflurane/oxygen mixture and fur removed from the ear and neck with depilatory cream (Boots the Chemist, UK). The mouse was placed on its side in the imaging so that both the ear and the draining Lymph node were facing the camera and light source. Imaging was performed at intervals following administration of fluorescently labeled ISCOMATRIX-647 and/or antigen (OVA-790). Band-pass excitation filters of 630 ± 10 nm was used for the visualization of ISCOMATRIX-647 and 770 ± 10 nm for OVA-790; emission filters were 700 ± 17.5 nm and 830 ± 17.5 nm respectively. The exposure time for fluorescent images was 120 s, with 1 × 1 pixel binning and an aperture f-stop of 2.51. X-Ray images were taken using a 0.4 mm aluminum filter with an exposure time of 60 s, 1 × 1 pixel binning and an aperture f-stop of 3.99. The entire acquisition time per animal was ~10 min. Image processing and analysis were performed using the Carestream molecular Imaging software (Carestream Health, Inc., Rochester, NY). Fluorescent images are displayed as pseudo-colored overlaid on the x-ray images. Scaling was determined manually and applied by the imaging software to the acquired gray-scale fluorescent images. Quantification of the fluorescence signal in the ear and the draining lymph node was determined using a region of interest (ROI) tool to carefully draw around the ear and around the location of the lymph node. The total fluorescence intensity (FI) in arbitrary units for each ROI was then determined by the software.

### Photoconversion of kaede mouse footpad

Photo-conversion of the Kaede mouse tissue was performed using a small mains operated 12x S06J bluray diode with a 405-G-2-glass lens (DTR's Laser Shop). The 405 nm laser diode operates at 600–650 mW, the emission spectra lies at 405 nm. Immediately prior too or at various times post-immunization (as detailed in the figure legend) mice were anesthetized and the ventral and dorsal sides of the tissue (hind paw) was illuminated on both sides with the laser for three times 5 s bursts. A 3 s interval between each burst was included to avoid tissue damage. The tissue was only photo-converted once per animal.

### *In vivo* labeling of SCS macrophages

eFluor660 or PE conjugated anti-CD169 (clone SER4) or Isotype control (clone) were purchased from eBiosciences, azide was removed by diafiltration and the antibody concentrated to ~1 mg/mL. 24 h prior to immunization 0.5 μg of antibody in 10 μL was injected into the ear pinnae (for flow cytometery experiments) or in 25 μL into the footpad (for imaging experiments).

### Imagestream analysis

Single cell suspensions were prepared from lymph nodes as described above for flow cytometry. Cells where stained with anti-CD11b (M1/70) conjugated to AlexaFluor 488 and anti-CD64 (X54-5/7.1) conjugated to Brilliant Violet 421 diluted in FcBlock. Samples where acquired on an Amnis ImageStream X MKII (EMD Millipore) equipped with 405, 488, 561, and 642 nm lasers and 6 detector channels using the 40 x objective. Data was analyzed using the IDEAS^TM^ software (EMD Millipore).

### *In vivo* multiphoton imaging

Multiphoton imaging was performed with a Zeiss LSM7 MP system equipped with both a 10x/0.3 NA air and a 20x/1.0NA water immersion objective lens (Zeiss) and a tuneable titanium/sapphire solid state 2-photon excitation source (Chamelon Ultra II; Coherent Laser Group). For *in vivo* imaging, animals were anesthetized with 3% isoflurane in 1.5 L/min oxygen, anesthesia was maintained with the isoflurane at 1.5–2%, and oxygen at 1.5 L/min. Core body temperature was continuously monitored and maintained by a thermostatically controlled heat mat. The popliteal was surgically exposed and the leg fixed in place using surgical veterinary glue (Vetbond; 3M). The hind quarters of the mouse was submerged in PBS warmed and maintained at 35–37°C throughout the experiment (45). Videos were acquired for 15–30 min at an X-Y pixel resolution 512 × 512 with 2 μm increments in Z. Images were processed using Volocity 5.5 (Perkin Elmer) after correction for tissue drift.

### Immunohistochemistry

Ear draining cervical lymph nodes were harvested and placed in OCT cryomedia (Tissue-Tek) and frozen, 8 μm sections were cut and placed on glass slides, sections were briefly fixed in acetone, rehydrated in PBS, nonspecific antibody binding was blocked using 2.4G2 culture supernatant containing 0.01% sodium azide, 5% mouse serum), followed 5% Normal Horse Serum in PBS (Vector Laboratories) sections were stained with anti-mouse-CD169 PE (BioLegend, clone:-3D6.112) antibodies at 1:100 overnight at 2–8°C in a humidity chamber. Sections were washed and coverslips applied using ProLong Gold Antifade (LifeTechnologies) to preserve fluorescence. Images were acquired using EVOS® FL Auto fluorescent microscope and imaging system (LifeTechnologies) at 10X magnification using the RFP detector. Final image processing was performed using ImageJ software.

### Statistics

Intergroup significance was determined by either a 1-way ANOVA or a 2-way ANOVA using GraphPad Prism 6 (GraphPad Software Inc, La Jolla, USA). A value of *P* ≤ 0.05 was considered significant.

## Author contributions

HES designed the research, performed the experiments, constructed the figures, analysed the data and wrote the manuscript. GRM provided technical assistance and contributed to writing the paper. AJH provided technical assistance and helped in design of the Kaede experiments. RAB provided technical assistance and helped in design of the injection site ablation experiments. EW provided technical assistance. AW, EM, ABM, JMB, MT and PG designed the research and contributed to writing the paper.

### Conflict of interest statement

AW, EM, and ABM are employees of CSL Limited. The remaining authors declare that the research was conducted in the absence of any commercial or financial relationships that could be construed as a potential conflict of interest.

## Supplementary material

Supplementary Figure 1 SCS macrophage debris do not appear to be taken up by CD11c+ DC. MFI of CD169 *ex vivo* on CD11b-CD11c+ DC at 4 **(A)** and 24 **(B)** h following immunization with PBS or 1 IU ISCOMATRIX and on CD11b+CD64+ monocytes at 24 h **(C)** following immunization with 1 IU ISCOMATRIX. ^****^*P* < 0.0001. IMX, ISCOMATRIX™ adjuvant.

Supplementary Figure 2 Detecting antigen presentation using the Yae-Eα system. OVA-Eα is taken and processed up by the antigen presenting cell. The resulting peptide antigens including Eα are loaded into MHCII and presented on the cell surface to CD4+ T cells. The YAe antibody binds to the peptide-MHCII complex essentially “seeing what the TCR sees,” and may be detected by flow cytometry providing a direct measure of antigen presentation.

Supplementary Figure 3 In the draining lymph nodes of LysM-EGFP mice treated with ISCOMATRIX GFP+ monocytes express MHCII while GFP+ neutrophils do not. LysM GFP mice were treated with 1 IU ISCOMATRIX in the footpad and the draining popliteal lymph node was harvested and analyzed by flow cytometry for the expression of MHCII on neutrophils and monocytes. **(A)** A representative FACS plot of Ly6G and CD64 expression on GFP+ cells. Neutrophils are considered Ly6G hi while Monocytes are CD64+**(B)** A representative histogram overlay shows the relative expression of GFP in neutrophils and monocytes following ISCOMATRIX treatment. **(C)** Median fluorescence intensity of MHCII on monocytes and neutrophils in the draining lymph node following ISCOMATRIX treatment. IMX, ISCOMATRIX™ adjuvant.

Supplementary Video 1 Following immunization with OVA/ISCOMATRIX T cells can be observed interacting with monocytes. OTII DSRed T cells (red) can be observed interacting (white) with GFP+ monocytes (green) in the popliteal lymph node at 20 h post-immunization with OVA/ISCOMATRIX. Scale bar = 100 μm.
