# Supplementary figures and images for: A Novel Cellular Pathway of Antigen Presentation and CD4 T Cell Activation in vivo
Source: Front Immunol. 2018 Nov 22;9:2684. doi: 10.3389/fimmu.2018.02684 (PMC6262026; doi:10.3389/fimmu.2018.02684)

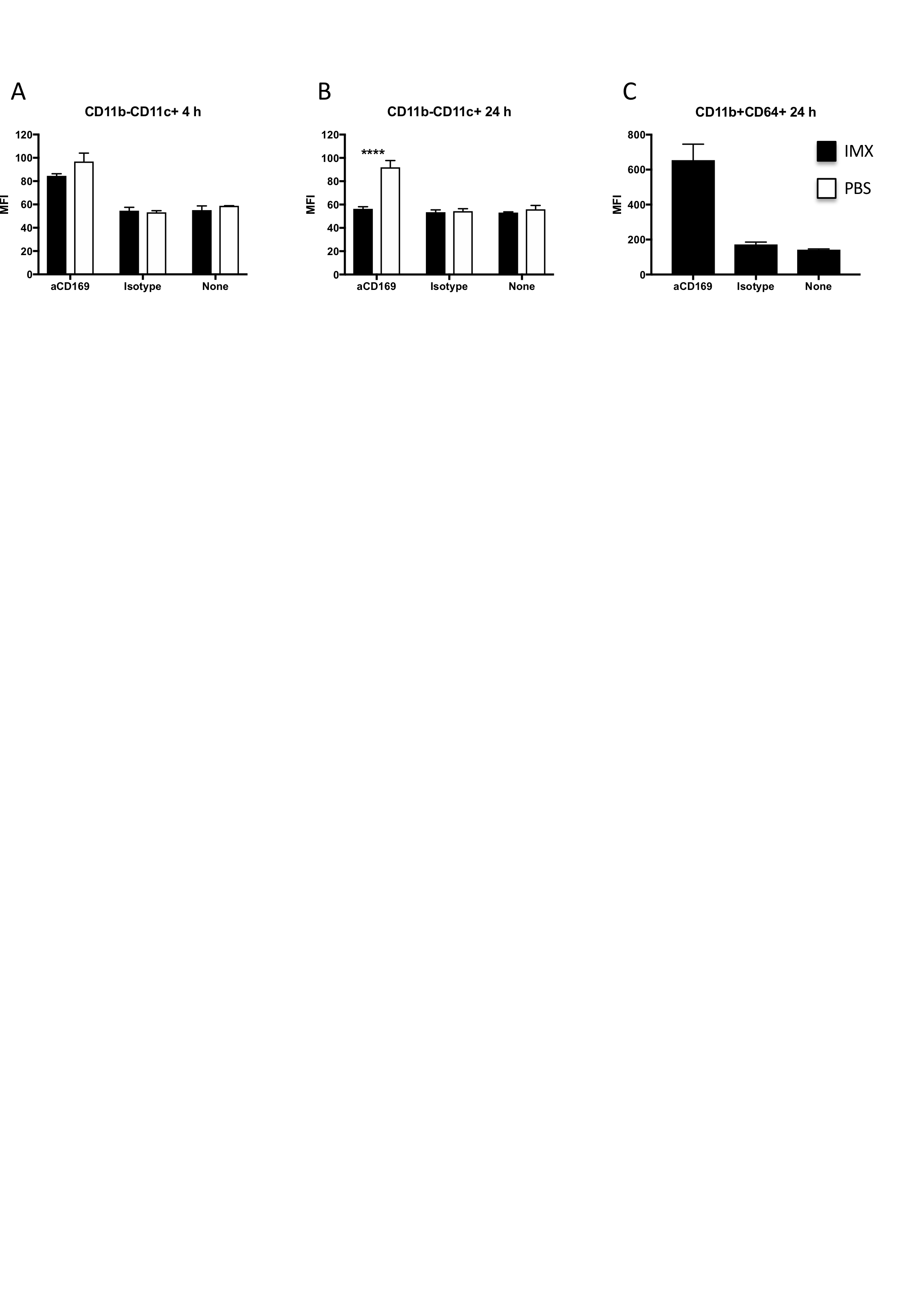

Supplement: Supplementary Figure 1 — SCS macrophage debris do not appear to be taken up by CD11c+ DC. MFI of CD169 ex vivo on CD11b-CD11c+ DC at 4 (A) and 24 (B) h following immunization with PBS or 1 IU ISCOMATRIX and on CD11b+CD64+ monocytes at 24 h (C) following immunization with 1 IU ISCOMATRIX. ****P < 0.0001. IMX, ISCOMATRIX™ adjuvant. [file Image_1.tiff]

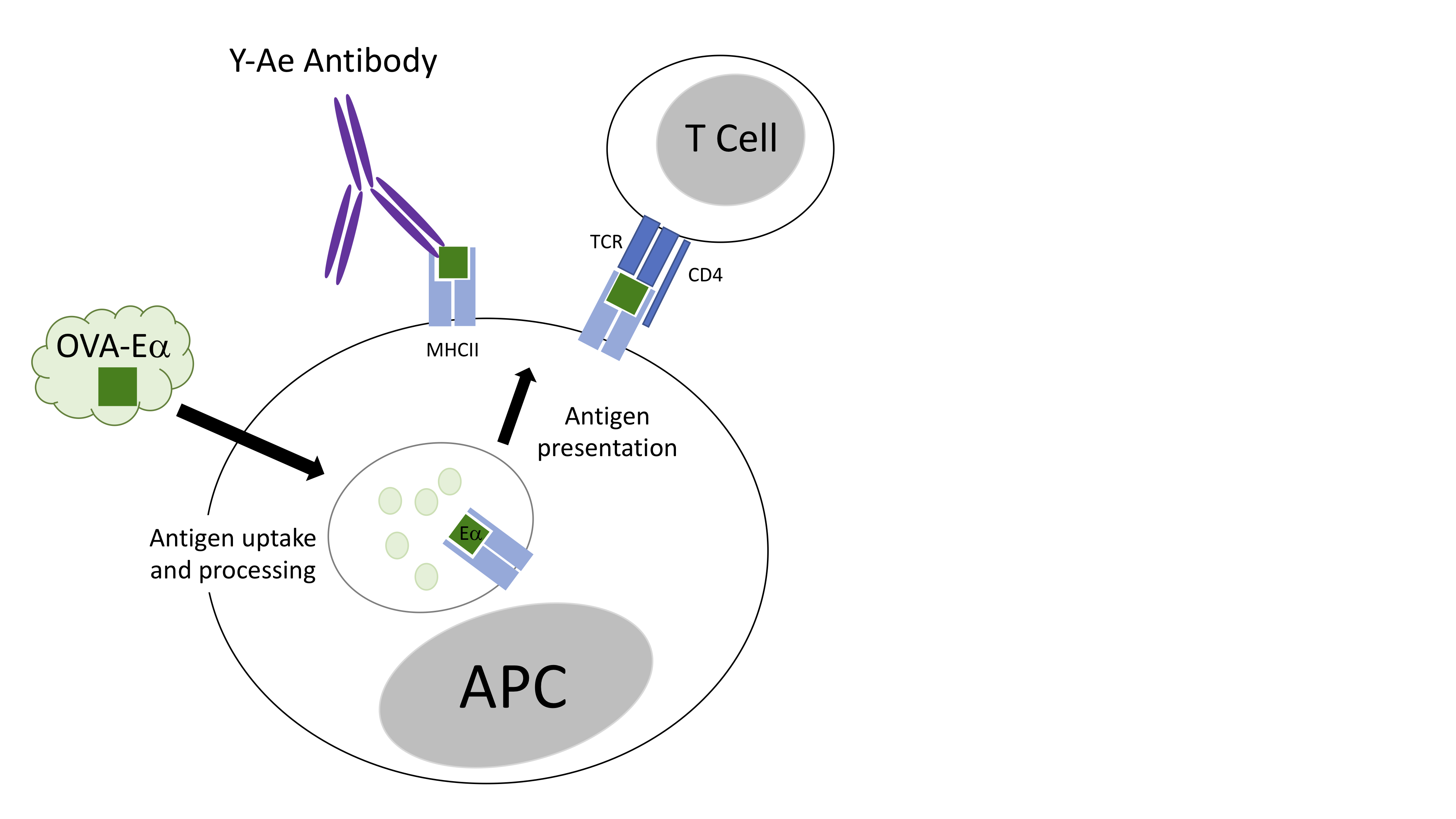

Supplement: Supplementary Figure 2 — Detecting antigen presentation using the Yae-Eα system. OVA-Eα is taken and processed up by the antigen presenting cell. The resulting peptide antigens including Eα are loaded into MHCII and presented on the cell surface to CD4+ T cells. The YAe antibody binds to the peptide-MHCII complex essentially “seeing what the TCR sees,” and may be detected by flow cytometry providing a direct measure of antigen presentation. [file Image_2.tiff]

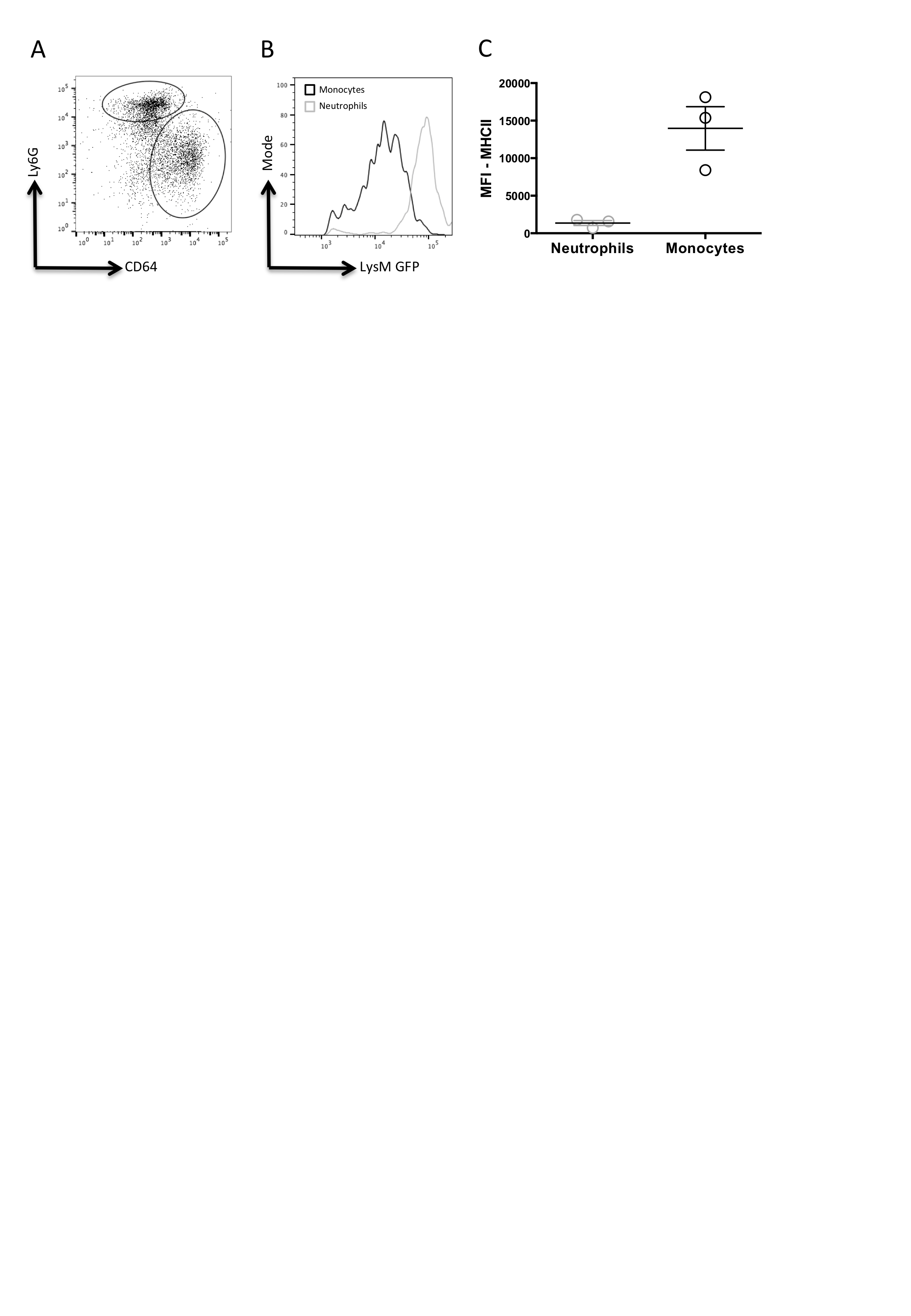

Supplement: Supplementary Figure 3 — In the draining lymph nodes of LysM-EGFP mice treated with ISCOMATRIX GFP+ monocytes express MHCII while GFP+ neutrophils do not. LysM GFP mice were treated with 1 IU ISCOMATRIX in the footpad and the draining popliteal lymph node was harvested and analyzed by flow cytometry for the expression of MHCII on neutrophils and monocytes. (A) A representative FACS plot of Ly6G and CD64 expression on GFP+ cells. Neutrophils are considered Ly6G hi while Monocytes are CD64+(B) A representative histogram overlay shows the relative expression of GFP in neutrophils and monocytes following ISCOMATRIX treatment. (C) Median fluorescence intensity of MHCII on monocytes and neutrophils in the draining lymph node following ISCOMATRIX treatment. IMX, ISCOMATRIX™ adjuvant. [file Image_3.tiff]
